# Influenza Virus Non-Structural Protein 1 (NS1) Disrupts Interferon Signaling

**DOI:** 10.1371/journal.pone.0013927

**Published:** 2010-11-10

**Authors:** Danlin Jia, Ramtin Rahbar, Renee W. Y. Chan, Suki M. Y. Lee, Michael C. W. Chan, Ben Xuhao Wang, Darren P. Baker, Bing Sun, J. S. Malik Peiris, John M. Nicholls, Eleanor N. Fish

**Affiliations:** 1 Department of Immunology, University of Toronto, Toronto, Canada; 2 Department of Pathology, University of Hong Kong, Hong Kong, People's Republic of China; 3 Department of Microbiology, University of Hong Kong, Hong Kong, People's Republic of China; 4 Biogen Idec Inc., Cambridge, Massachusetts, United States of America; 5 Shanghai Institutes for Biological Sciences, Chinese Academy of Sciences, Shanghai, People's Republic of China; 6 Division of Cell and Molecular Biology, Toronto General Research Institute, University Health Network, Toronto, Canada; Tsinghua University, China

## Abstract

Type I interferons (IFNs) function as the first line of defense against viral infections by modulating cell growth, establishing an antiviral state and influencing the activation of various immune cells. Viruses such as influenza have developed mechanisms to evade this defense mechanism and during infection with influenza A viruses, the non-structural protein 1 (NS1) encoded by the virus genome suppresses induction of IFNs-α/β. Here we show that expression of avian H5N1 NS1 in HeLa cells leads to a block in IFN signaling. H5N1 NS1 reduces IFN-inducible tyrosine phosphorylation of STAT1, STAT2 and STAT3 and inhibits the nuclear translocation of phospho-STAT2 and the formation of IFN-inducible STAT1:1-, STAT1:3- and STAT3:3- DNA complexes. Inhibition of IFN-inducible STAT signaling by NS1 in HeLa cells is, in part, a consequence of NS1-mediated inhibition of expression of the IFN receptor subunit, IFNAR1. In support of this NS1-mediated inhibition, we observed a reduction in expression of *ifnar1* in *ex vivo* human non-tumor lung tissues infected with H5N1 and H1N1 viruses. Moreover, H1N1 and H5N1 virus infection of human monocyte-derived macrophages led to inhibition of both *ifnar1* and *ifnar2* expression. In addition, NS1 expression induces up-regulation of the JAK/STAT inhibitors, SOCS1 and SOCS3. By contrast, treatment of *ex vivo* human lung tissues with IFN-α results in the up-regulation of a number of IFN-stimulated genes and inhibits both H5N1 and H1N1 virus replication. The data suggest that NS1 can directly interfere with IFN signaling to enhance viral replication, but that treatment with IFN can nevertheless override these inhibitory effects to block H5N1 and H1N1 virus infections.

## Introduction

Transcriptional activation of IFNs-α/β is rapidly initiated in response to detection of viral-derived factors by cellular pattern recognition receptors [1]. IFNs-α/β subsequently bind their cognate cell surface receptor, leading to the activation of the receptor-associated kinases, Jak1 and Tyk2 [2]. Signal transducers and activators of transcription (STAT) proteins are recruited to the receptor, phosphorylated on tyrosine residues by these Jaks, then released from the receptor to form transcription factor complexes that translocate into the nucleus and upregulate the expression of IFN-stimulated genes (ISG). IFN signaling can be negatively regulated by members of the suppressors of cytokine signaling (SOCS) family. SOCS1 has been shown to block IFN signaling through direct physical binding with Jak1, whereas SOCS3 and CIS can interact with the phosphorylated receptor to prevent the recruitment and phosphorylation of downstream mediators like STAT proteins [2].

Given the critical role of IFNs-α/β as a first line of defense against infection, it is not surprising that many viruses have evolved strategies to block an IFN response as a means to increase their replication efficiency [2], [3]. Viral-mediated inhibition of IFNs can be generalized into three categories, including disruption of IFN induction, disruption of IFN-inducible signaling and disruption of IFN-mediated effector functions.

The non-structural protein 1 (NS1) of influenza A viruses exerts its inhibitory effects on IFN predominately by interfering with IFN production [4]. NS1 disrupts the induction of IFNs by first inhibiting the intracellular sensor RIG-I, which plays a critical role in detecting ssRNA during influenza A virus infection [5]. RIG-I activation leads to association with the downstream adaptor IPS-1, resulting in phosphorylation of IRF3 and subsequent transcriptional activation of IFN-β [5], [6]. Experimental evidence suggests that NS1 can associate with RIG-I, as well as TRIM25, a ubiquitin ligase required for RIG-I activation, to prevent its downstream activation of the IFN-β promoter [7], [8]. Both IRF3 translocation and NFκB activation are impaired in the presence of NS1, which in turn blocks the induction of proinflammatory cytokines and IFNs [9], [10]. In addition, NS1 can interfere with host mRNA splicing and polyadenylation by interacting with U6 snRNA and the cleavage polyadenylation specificity factor 30 (CPSF30), respectively. Notably, in addition to inhibition of IFN-β gene transcription, NS1 promotes the accumulation of IFN-β pre-mRNA transcripts [11].

NS1 can activate phosphoinositide 3-kinase (PI3K) by interacting with the regulatory subunit, p85, through a putative SH2-binding domain. Activation of PI3K by NS1 leads to the downstream activation of Akt, and delays apoptosis of influenza virus-infected cells [12], [13]. Given that NS1 has been shown to modulate intracellular signaling events and inhibit the induction of IFN, we undertook experiments to determine whether avian H5N1 influenza NS1 can also influence facets of IFN-α/β-inducible signaling. In addition, as more influenza A viruses, including the highly pathogenic avian H5N1 strain and the circulating swine origin H1N1 pandemic 2009 strain (S-OIV, H1N1pdm) are developing resistance to the antiviral agents oseltamivir and/or the adamantine derivatives, there is an urgent need for alternative antiviral therapies [14], [15], [16]. Accordingly we examined the therapeutic potential of the synthetic IFN-α, IFN alfacon-1, as an antiviral against H5N1 and H1N1 influenza A infections, employing a novel human non-tumor lung tissue explant model.

We provide evidence that expression of H5N1 NS1 reduces IFN-inducible phosphorylation of STAT proteins and results in decreased formation of downstream STAT:DNA complexes. We attribute this NS1-medited inhibition of IFN-inducible signaling to the effects of NS1 on restricting the cell surface expression of IFN receptor expression, and also upregulation of the signaling inhibitors SOCS1 and SOCS3. We provide evidence that treatment of human lung tissue with IFN alfacon-1 inhibits both H1N1 and H5N1 viral replication, overriding the inhibitory effects of NS1.

## Materials and Methods

### Cells and reagents

The human cervical carcinoma cell line HeLa was obtained from ATCC (Manassas, VA). Cells were cultured in Dulbecco's modified Eagle's medium (Invitrogen), supplemented with 10% fetal calf serum (FCS), 100 U/mL penicillin, 100 µg/mL streptomycin (Invitrogen). Plasmids pBudCE4.1 and pBudCE4.1-H5N1 NS1-HA (A/Duck/Hubei/L-1) were kindly provided by Dr. Bing Sun. Monocyte-derived macrophages were produced, as described previously [17]. Fresh lung biopsies were obtained from non-tumor lung tissue obtained during surgical resection of lung tissue at Queen Mary Hospital in Hong Kong. Written, informed consent was obtained from all patients and institutional approval was granted by The Hong Kong University and Hospital Authority (Hong Kong West) Institutional Review Board. The biopsies or tissue fragments were excess to the requirements of clinical diagnosis. Lung tissue from each donor was cut into multiple fragments (2–3 mm). The tissues were immediately placed into culture medium (F-12K nutrient mixture with L-glutamine, and antibiotics) and infected with either influenza A H5N1 (A/Vietnam/3046/04 or A/HK/483/97) or H1N1 (A/HK/54/98 or A/Ca/04/09 (H1N1pdm)) viruses within three hours of collection. Virus was adsorbed for one hour at 37°C, then free virus was removed by washing the tissue fragments in warm PBS. Biopsies with no virus added were used as controls. The biopsy or tissue fragments were incubated at 37°C for the indicated times.

Human recombinant IFN alfacon-1 (IFN alfacon-1, specific activity, 6×10^8^ U/mL) was provided by Three Rivers Pharmaceuticals (Pittsburgh, PA). Human IFN-β (IFN β-1a, specific activity, 1.2×10^7^ U/mL) was provided by BiogenIdec Inc., Cambridge, MA. Antibodies against p-STAT1, p-STAT2, p-STAT3, STAT1, STAT3, HA, SOCS1, and SOCS3 were purchased from Cell signaling. Antibodies against STAT2 and β-actin were obtained from Santa Cruz Biotechnology (Santa Cruz, CA).

### Transfection and virus infections

Cells (2×10^5^) were transfected using Lipofectamine LTX (Invitrogen, CA) according to the manufacturer's protocol. Briefly, cells were seeded in 6 well plates 24 hours before transfection. Plasmid DNA and transfection reagent were mixed in serum-free medium and incubated for 30 minutes at room temperature. Transfection complexes were then gently added into individual wells of the 6-well plate.

For influenza A infection of primary lung tissues, virus was used at a titer of 1×10^7.3^TCID_50_/mL for H5N1, 1×10^6.4^TCID_50_/mL for A/HK/54/98 H1N1 and 1×10^7^TCID_50_/mL for pandemic A/Ca/04/09 H1N1. Human monocyte-derived macrophages were infected with A/HK/483/97 H5N1 or H1N1 at an MOI of 2, or mock infected.

### Measurements of viral infectivity

The extent of viral infection was determined using a TCID_50_ (50% tissue culture infectious doses per mL) assay. Briefly, a confluent 96-well plate of MDCK cells was prepared one day prior to the viral titration. Cells were then washed with PBS and overlayed with serum-free Minimum Essential Medium (MEM) supplemented with 100 U/mL penicillin and 100 µg/mL streptomycin and 2 µg/mL of TPCK (tosylsulfonyl phenylalanylchloromethyl ketone) treated trypsin. 1∶10 serial dilutions of stock virus were adsorbed onto the plates in quadruplicate. The plates were observed for cytopathic effect daily. The end-point of viral dilution leading to CPE in 50% of inoculated wells was estimated using the Karber method [18].

### Immunoblotting and immunoprecipitation

Cells were lysed in 30 µL of lysis buffer (1% Triton X-100, 0.5% Nonidet P-40, 150 mM NaCl, 10 mM Tris-HCl, pH 7.4, 1 mM EDTA, 1 mM EGTA, 0.2 mM phenylmethylsulfonyl fluoride). Protein concentration was determined using the Bio-Rad protein DC assay kit (Bio-Rad Laboratories, Hercules, CA). 30 µg of protein lysate/sample was denatured in 5× sample reducing buffer and resolved by SDS-PAGE. The separated proteins were transferred to a nitrocellulose membrane followed by blocking with 5% bovine serum albumin (w/v) in TBS-T for 1 h at room temperature. Membranes were probed with the indicated specified antibodies. Proteins were visualized using the ECL detection system (Pierce, Rockford, IL). For immunoprecipitation assays, cells transfected with vector alone or with H5N1 NS1 plasmid were incubated in hypotonic lysis buffer containing 10 mM HEPES, pH 7.4 for 30 minutes on ice, and the suspension was then briefly sonicated. The suspension was centrifuged at 14000 rpm for 30 min at 4°C. The supernatant was collected and protein concentration measured using the Bio-Rad protein assay kit. 500 µg of protein was incubated with 1 µg of either anti-STAT1 antibody, anti-STAT2 antibody or IgG isotype control antibody. Antibodies were immunoprecipitated with protein A/G-Sepharose beads (Santa Cruz Biotechnology) and washed 6 times with pH 7.4 HEPES buffer. Beads were denatured in 5× sample reducing buffer and resolved by SDS-PAGE.

### Cell sorting and flow cytometric analysis for IFNAR1 and IFNAR2 cell surface expression

24 hours post-transfection, cells were first washed with PBS and subsequently incubated with Versene for 15 minutes. Suspended cells were collected and centrifuged at 1500 rpm for 5 minutes. Cell pellets were then resuspended at 1×10^7^ cells/mL in 2% FCS. 5×10^5^ GFP^+^ sorted cells were incubated with either anti-human IFNAR1 (BiogenIdec)[19] or anti-human IFNAR2 antibody (Caltag), followed by PE-conjugated anti-mouse IgG antibody. As isotype controls, cells were incubated with PE-labeled isotype control IgG antibody (eBioscience). All analyses were performed using the FACS Calibur and CellQuest software. Cells were gated based on forward and side scatter.

### RNA extraction and cDNA synthesis

Cells were lysed using buffer RLT + β-mercaptoethanol with Qiagen QIA-shredder columns. RNA isolations were carried out using Qiagen RNeasy mini kits, according to the manufacturer's protocol, with DNA digestion. Total cellular RNA was eluted in RNase-free water. The concentration of RNA was determined by UV spectrophotometry at 260 nm wavelength (Beckman). cDNAs were synthesized using 0.5 µg RNA and oligo-dT primers and Superscript III reverse transcriptase according to the manufacturer's protocol (Invitrogen, CA).

### Real-time polymerase chain reaction (RT-PCR)

Real-time PCR was carried out using a LightCycler® (Roche) in conjunction with LightCycler®FastStart DNA Master SYBR Green PLUS I Kits (Roche). Reactions were carried out in 20 µL volumes containing 4 µL Master SYBR Green PLUS buffer at a final concentration of 1X, 5 µL of 0.1 µg/µL cDNA. 9 µL of PCR-grade water and 1 µL of each 20 µM forward and reverse primers. PCR reactions were carried out under the following conditions: pre-incubation at 95°C for 10 minutes, followed by 45 amplification cycles of denaturation for 10 seconds, annealing for 5 seconds at 60°C, extension at 72°C for 10 seconds, melting curve analysis at 65°C for 15 seconds and a continuous acquisition mode of 95°C with a temperature transition rate of 0.1°C/s. The data were subsequently analyzed using software RealQuant. PCRs were carried using the following primers:

*Ifnar1*

(forward) 5′ CACTGACTGTATATTGTGTGAAAGCCAGAG 3′

(reverse) 5′ CATCTATACTGGAAGAAGGTTTAAGTGATG 3′

*Ifnar2*

(forward) 5′ ATTTCCGGTCCATCTTATCAT 3′

(reverse) 5′ACTGAACAACGTTGTGTTCC 3′

Influenza A *m* gene

(forward) 5′ CTTCTAACCGAGGTCGAAACG 3′

(reverse) 5′ GGCATTTTGGACAAAGCGTCTA 3′

*Isg*15

(forward) 5′ TCCTGGTGAGGAATAACAAGGG 3′

(reverse) 5′ CTCAGCCAGAACAGGTCGTC 3′

*pkr*

(forward) 5′ GCCTTTTCATCCAAATGGAATTC 3′

(reverse) 5′ GAAATCTGTTCTGGGCTCATG 3′

2′5′-*oas*

(forward) 5′ AGCTTCATGGAGAGGGGCA 3′

(reverse) 5′ AGGCCTGGCTGAATTACCCAT 3′

*socs1*

(forward) 5′ TTGCCTGGA ACCATGTGG 3′

(reverse) 5′ GGTCCTGGCCTCCAGATACAG 3′

*socs3*

(forward) 5′ GGAGTTCCTGGACCAGTACG 3′

(reverse) 5′ TTCTTGTGCTTGTGCCATGT 3′

*β* *-actin*

(forward) 5′ACATGGAGAAAAATCTGGCAC 3′

(reverse) 5′ GTAGCACAGCTTCTCCTTAATGT 3′

### Electrophoretic mobility shift assay

10 µg of nuclear protein from untreated or IFN-treated cells was extracted as described previously [20]. Extracts were incubated with 1 µg poly(dI-dC)poly(dI-dC) for 10 minutes at 4°C in buffer containing 60 mM EGTA, and 5% Ficoll (final volume 30 µL). 40,000 counts per minute (cpm) of radiolabeled sis-inducible element (SIE) (5′ AGCTTCATTTCCCGTAAATCCCT 3′) were added and the reaction mixture was incubated for an additional 20 minutes at ambient temperature. Protein-DNA complexes were resolved on a 4.5% polyacrylamide gel using 0.5× TBE (final concentration 45 mM Tris borate, 1 mM EDTA) as the running buffer. Gels were dried and exposed to autoradiographic film (Kodak BioMax MS) overnight at −80°C. A supershift assay to identify specific STAT:SIE complexes was carried out prior to EMSA by incubating nuclear extracts with 2 µg of anti-STAT1 (C-136X (batch #s L1004, B0403), Santa Cruz Biotechnology) or anti-STAT3 (H-190X, Santa Cruz Biotechnology; 9132, Cell Signaling Technology) antibodies for 30 minutes prior to addition of the radiolabeled SIE for a further 20 minutes.

### Immunohistochemistry and confocal microscopy

Cells transfected with H5N1 NS1 plasmid were stained as described previously [21]. Various proteins were visualized using fluorescence-conjugated secondary antibodies (Alexafluor-488: green, Alexafluor-555: red and Alexafluor-647: blue) (Amersham Biosciences, Cardiff, UK). Images were collected using an upright Leica SP2 confocal laser-scanning microscope (Leica Microsystems Heidelberg GmbH, Mannheim, Germany), a 100× oil immersion lens (1.4 numerical aperture), and a 4× digital zoom. Laser excitations were 488 nm (Ar/Kr) and 543 nm (He/Ne), attenuated to 10% and 50%, respectively, by way of an acoustic-optical transmission filter. Sequential scan mode was used to eliminate cross talk of detected signals, which were filtered between 500 to 530 nm and 560 to 660 nm. Image resolution was 512 dpi by 512 dpi (12 bit), and line averaging (4×) was used. Optical sections were collected at 0.5 µm intervals through the entire cell.

To stain for influenza nucleoprotein, lung tissue explants were fixed in 10% neutral buffered formalin and processed for paraffin embedding and immunohistochemistry using a mouse anti-influenza nucleoprotein antibody (HB65, EVL Laboratories, The Netherlands).

## Results

### H5N1 NS1 expression inhibits IFN-inducible STAT phosphorylation

**Figure 1:**
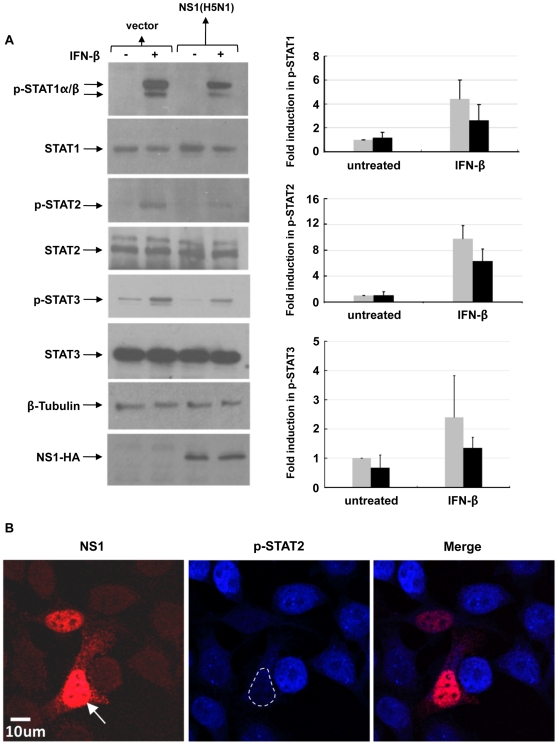
H5N1 NS1 expression inhibits IFN-inducible STAT phosphorylation. **A**) HeLa cells transfected with vector alone (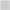) or HA-tagged NS1 plasmid (▪) were left untreated (−) or treated (+) with IFN-β (1×103 U/mL) for 15 mins, 24 hr post-transfection. Cells were harvested, lysates were resolved by SDS-PAGE and immunoblotted with the indicated anti-phospho-STAT1, anti-phospho-STAT2, anti-phospho-STAT3 and anti-HA(NS1) antibodies. Membranes were stripped and reprobed with anti-STAT1, anti-STAT2, anti-STAT3 and anti-β-tubulin antibodies as loading controls. Relative fold induction of phosphorylated STAT proteins was calculated using signal intensity of phospho-STATs over total STATs and normalized with untreated, vector transfected cells. The data plots are representative of three independent experiments. **B**) HeLa cells transfected with HA-tagged NS1 plasmid were treated with IFN-β (1×10^3^ U/mL) for 15 mins. Cells were then fixed and stained for HA (red) and phospho-STAT2 (blue), and analyzed by confocal microscopy as described in Materials and Methods. The white arrow identifies the nuclear predominance of NS1and the broken line in the middle panel defines the nucleus showing reduced phospho-STAT2. Data are representative of two independent experiments.

To investigate the effects of H5N1 NS1 expression on IFN signaling, HeLa cells were transfected with either vector alone or plasmid containing HA-tagged H5N1 NS1. 24 hours post-transfection, cells were treated with IFN-β (1×10^3^ U/mL) to promote IFN-inducible signaling. In contrast to cells transfected with vector alone, there was a notable reduction in IFN-inducible STAT1, STAT2 and STAT3 phosphorylation in cells expressing H5N1 NS1 (Figure 1A). Human lung epithelial A549 cells transfected with H5N1 NS1 likewise exhibit a reduction in IFN-β-inducible STAT1 phosphorylation (data not shown).

To confirm that the inhibition of IFN-inducible STAT phosphorylation is a consequence of NS1 expression, in an identical series of transfection experiments, cells were fixed and stained with both anti-phospho-STAT2 and anti-HA (NS1) antibodies. Confocal microscopy revealed that, in contrast to cells lacking H5N1 NS1 expression, that exhibit strong IFN-inducible phospho-STAT2 staining in their nuclei, there is a notable reduction in IFN-inducible phospho-STAT2 staining in H5N1 NS1-expressing cells (Figure 1B).

### NS1 inhibits IFN-inducible STAT-DNA binding

**Figure 2:**
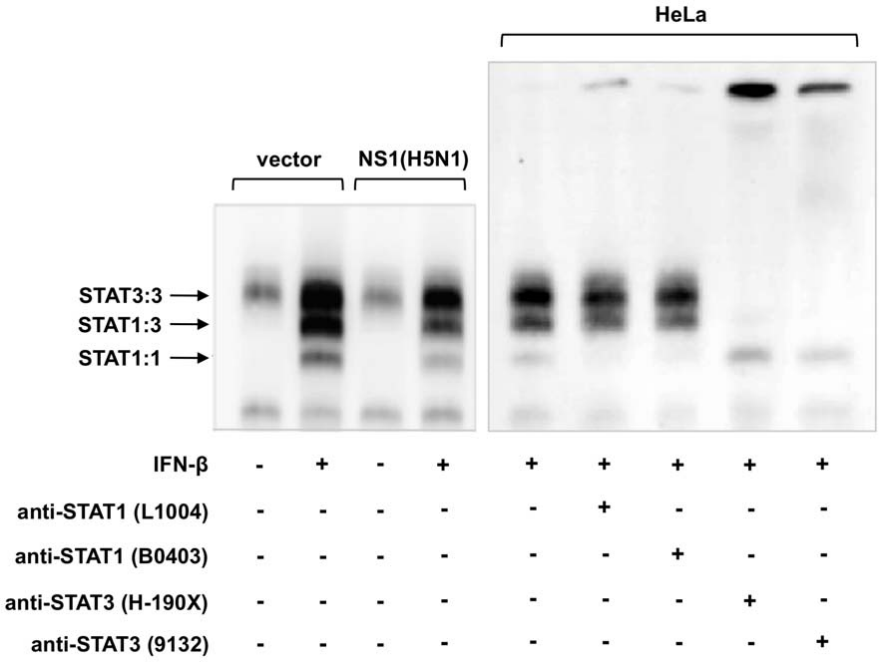
H5N1 NS1 expression inhibits IFN-inducible STAT:SIE complex formation. **A**) HeLa cells transfected with either vector alone or HA-tagged NS1 plasmid were left untreated (−) or treated (+) with IFN-β (1×10^3^ U/mL) for 15 mins, 24 hr post-transfection. Nuclear extracts were isolated and equal amounts of protein were incubated with ^32^P-labeled SIE probe. Complexes were resolved by native gel electrophoresis and visualized by autoradiography. Data are representative of two independent experiments. HeLa cells were treated with IFN-β (1×10^3^ U/mL) for 15 mins. Nuclear extracts were isolated and equal amount of protein were incubated with ^32^P-labeled SIE probe in the presence or absence of 2 µg of anti-STAT1 and anti-STAT3 antibodies, as indicated. Complexes were resolved by native gel electrophoresis and visualized by autoradiography.

STAT proteins, once activated by phosphorylation, form functional homo- or hetero-dimeric complexes that migrate to the nucleus and target specific promoter elements to initiate transcriptional activation. To examine the functional consequence of H5N1 NS1-mediated inhibition of IFN-inducible STAT phosphorylation, we examined STAT-DNA binding, using electrophoretic mobility shift assay (EMSA) studies. HeLa cells were transfected with empty vector or vector containing cDNA encoding H5N1 NS1 for 24 hours, then cells were either left untreated or treated with IFN-β. Nuclear extracts were prepared and incubated with a radiolabeled sis-inducible element (SIE), then resolved by agarose gel electrophoresis to determine the formation of IFN-inducible STAT:SIE complexes. In contrast to cells transfected with empty vector, we observed a reduction in the characteristic STAT1:1:SIE, STAT1:3:SIE, and STAT3:3:SIE complexes in the presence of H5N1 NS1 protein (Figure 2). In further support of a functional consequence of NS1-mediated inhibition of IFN-inducible STAT phosphorylation, the effects of NS1 on IFN-inducible STAT2 activation were evident as reduced nuclear phospho-STAT2, described in Figure 1B.

### H5N1 and H1N1 infection affect IFNAR expression

**Figure 3:**
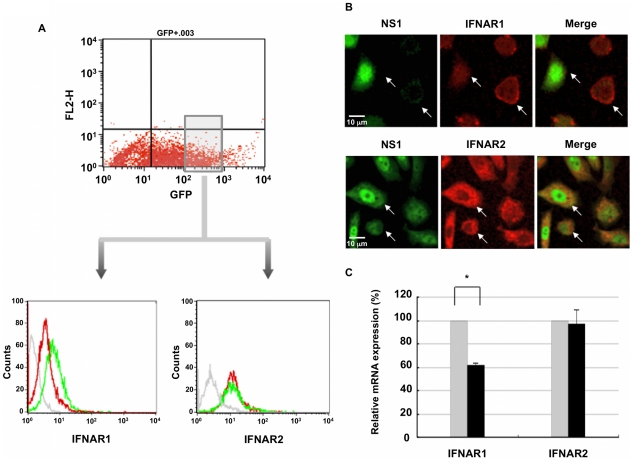
H5N1 NS1 reduces surface IFNAR1 but not IFNAR2 expression. **A**) HeLa cells were transfected with either GFP vector alone (green) or GFP vector containing HA-tagged NS1 (red), then 24 hr post-transfection, GFP-positive cells were FACS sorted and stained for IFNAR1 or IFNAR2 and analyzed by FACS. Data are representative of three independent experiments. **B**) HeLa cells were transfected with HA-tagged NS1 plasmid and 24 hr post-transfection were fixed and stained for HA (green) and either IFNAR1 (red; upper panel) or IFNAR2 (red; lower panel), and analyzed by confocal microscopy. Data are representative of two independent experiments. **C**) HeLa cells were transfected with either GFP vector alone (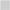) or GFP vector containing HA-tagged NS1 (▪). 24 hr post-transfection, GFP positive cells were FACS sorted, RNA extracted and cDNA synthesized. *Ifnar1*, *ifnar2* and *β*-actin gene expression were analyzed by RT-PCR. Gene expression was calculated relative to β-actin gene expression and normalized to cells transfected with GFP vector alone. Data are representative of two independent experiments. Significant differences (asterisk) were determined by Student's t-test (p<0.05).

Given the effects of NS1 on IFN-inducible activation of STATs, we next examined the influence of NS1 expression on upstream molecules involved in IFN signaling, namely the receptors, IFNAR1 and IFNAR2. HeLa cells were transfected with vector expressing GFP or a plasmid that co-expresses NS1 and GFP. GFP-positive cells were FACS sorted 24 hours post-transfection and analyzed for surface IFNAR1 and IFNAR2 expression using flow cytometry. Cells expressing H5N1 NS1 exhibited a reduced level of surface IFNAR1 when compared to cells expressing vector alone (Figure 3A). Notably, cell surface IFNAR2 expression was not affected by the expression of NS1. In a subsequent series of experiments we confirmed that this reduction in cell surface IFNAR1 expression was restricted to cells expressing NS1 (Figure 3B). To determine whether the differential surface expression of IFNAR1 and IFNAR2 in the presence of H5N1 NS1 is a consequence of regulation at the mRNA level, HeLa cells were transfected with either vector-GFP or the NS1-GFP plasmid as above, and GFP-positive cells were FACS sorted 24 hours following transfection for subsequent RNA analysis. *Ifnar1* and *ifnar2* gene expression were analyzed using RT-PCR. In contrast to vector-GFP transfected cells, we observed a reduction in *ifnar1* but not *ifnar2* gene expression in cells transfected with H5N1 NS1 (Figure 3C).

**Figure 4:**
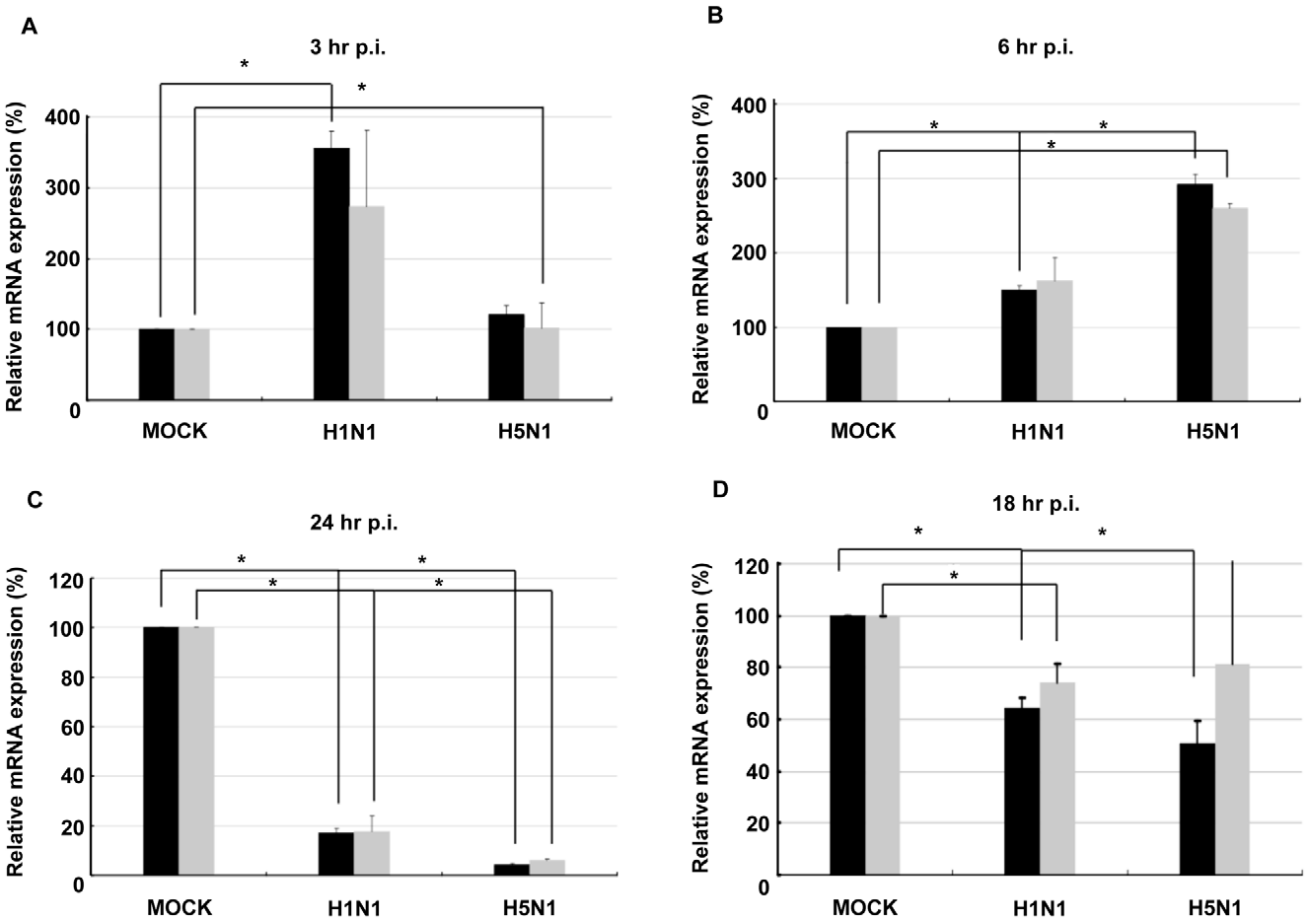
Influenza virus infection reduces *ifnar1* and *ifnar2* expression at 24 hours post-infection in human monocyte-derived macrophages and *ex vivo* lung tissues. Human monocyte-derived macrophages were infected with A/HK/483/97 H5N1 or A/HK/54/98 H1N1 virus (Multiplicity of Infection (MOI)  = 2). RNA was extracted from the cells at **A**) 3 hr, **B**) 6 hr, and **C**) 24 hr post-infection. Following cDNA synthesis, *ifnar1* (▪) and *ifnar2* (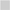) expression was assayed by real-time PCR. Data shown are fold induction of gene expression relative to mock-infected control after normalizing to β-actin in each sample. Representative data of duplicate experiments with means of triplicate assays are shown; **D**) Human lung explant tissue was either mock-infected (PBS) or infected with A/HK/483/97 H5N1 or A/HK/54/98 H1N1 influenza A viruses, as described in Materials & Methods. 18 hr post-infection tissue was processed to extract RNA. cDNA was synthesized and expression of *ifnar1*, *ifnar2* and *β-actin* gene expression was measured by RT-PCR analysis. Gene expression was calculated relative to *β-actin* gene expression and normalized to mock infected tissues. Data are representative of two independent experiments. Significant differences (asterisk) were determined by Student's t-test (p<0.05).

Within the lung, both pneumocytes as well as macrophages are targets for H5N1 and H1N1 infection. Accordingly, in the next series of experiments we employed human monocyte-derived macrophages to examine the effects of both H5N1 and H1N1 virus infections on IFNAR expression. We found that both *ifnar1* and *ifnar*2 were upregulated in response to H1N1 and H5N1 viruses within 6 hours post-infection compared to mock infection (Figure 4A, B), but that by 24 hours, there was a reduction of both *ifnar1* and *ifnar2* compared to mock infection (Figure 4C),

To investigate if inhibition of either *ifnar1* or *ifnar2* occurs in the context of influenza A infection in the intact lung, human lung explant tissues were infected with either H1N1 or H5N1 influenza A viruses. 18 hours post-infection, RNA was collected and analyzed. Infection with both viruses led to a selective reduction in *ifnar1* gene expression when compared to mock-infected control tissues (Figure 4D). Notably, infection with the H5N1 influenza A strain led to a greater reduction in *ifnar1* gene expression compared to infection with H1N1 virus. The modest inhibitory effects of H1N1 and H5N1 infection on *ifnar2* gene expression were not statistically significant.

### H5N1 and H1N1 regulate SOCS expression

**Figure 5:**
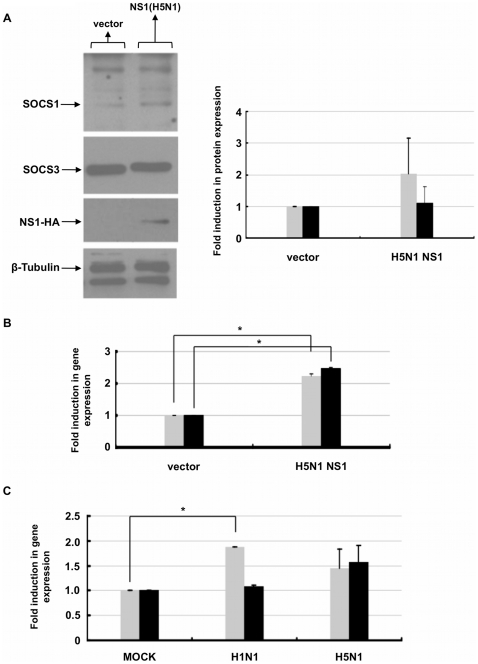
NS1 regulates SOCS1 and SOCS3 expression. **A**) HeLa cells were transfected with vector alone or HA-tagged NS1 plasmid, then 24 hr post-transfection cells were harvested and lysates were resolved by SDS-PAGE and immunoblotted with the indicated anti-SOCS1 and anti-SOCS3 antibodies. Membranes were probed with anti-HA antibody to confirm expression of NS1, and anti-tubulin antibody was applied as a loading control. Relative fold induction of proteins was calculated using signal intensity over loading and normalized against vector transfected cells (SOCS1 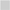, SOCS3 ▪). Data are representative of two independent experiments. **B**) HeLa cells were transfected with either GFP vector alone (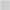) or GFP vector containing HA-tagged NS1 (▪). 24 hr post-transfection, GFP^+^ cells were sorted, RNA extracted and cDNA synthesized. Gene expression of *socs1* (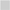), *socs3* (▪) and *β-actin* gene expression were analyzed by RT-PCR. Gene expression was calculated relative to β-actin gene expression and normalized to cells transfected with GFP vector alone. Data are representative of two independent experiments. **C**) RNA from human lung tissue either mock-infected (PBS) or infected with A/HK/54/98 H1N1 or H5N1 influenza A viruses was collected 18 hr post-infection. cDNA was synthesized and expression of *socs1*(▪), *socs3*(▪) and *β-actin* gene expression was measured by RT-PCR analysis. Gene expression was calculated relative to β-actin gene expression and normalized to mock infected cells. Data are representative of two independent experiments. Significant differences (asterisk) were determined by Student's t-test (p<0.05).

To determine the effect of H5N1 NS1 on negative regulators of type I IFN signaling, namely SOCS proteins, HeLa cells were transfected with plasmid containing the H5N1 *ns1* gene and 24 hours post-transfection protein extracts were processed and analyzed for SOCS1 and SOCS3 protein expression. In contrast to cells transfected with vector alone, we observed an increase in SOCS1 but not SOCS3 protein in cells expressing H5N1 *ns1* (Figure 5A). Interestingly, when we examined the gene expression profile of *socs1* and *socs3* in cells that co-express GFP and NS1, we observed a two-fold increase in both *socs1* and *socs3* gene expression when compared to control cells that express GFP alone (Figure 5B). Moreover, RT-PCR analysis of infected human lung tissue explants revealed that both *socs1* and *socs3* gene expression was upregulated by H5N1 infection, whereas only *socs1* gene expression was increased by H1N1 infection (Figure 5C).

### IFN treatment upregulates IFN sensitive genes and inhibits H5N1 and H1N1 influenza A replication in primary human lung cells

**Figure 6:**
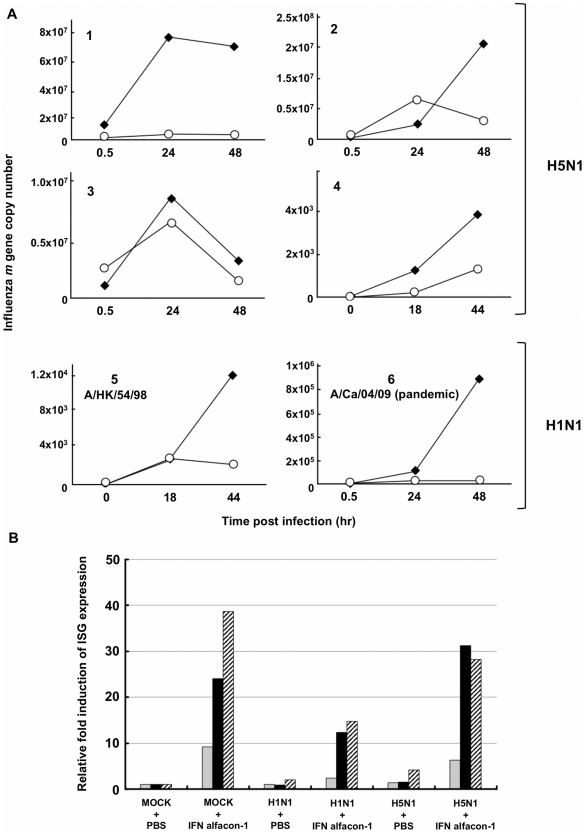
Treatment with IFN alfacon-1 inhibits H5N1 and H1N1 replication in primary human lung cells and upregulates ISG expression. Different human surgical lung tissue explants (1–6) were either left untreated (♦) or treated with IFN alfacon-1 (1.2×104 U/mL) (○) for 16 hr. Tissues were then infected with H5N1 (1–4) or H1N1 influenza A viruses (5, 6), as indicated. At different time points post infection, RNA from cells was collected and cDNA synthesized. Gene expression for **A**) influenza A *m* gene, was measured by RT-PCR. Gene expression for **B**) pkr (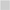), isg15 (▪), 2′5′-oas (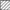), and β-actin, was measured by RT-PCR analysis at 18 hr post-infection with H5N1 or A/HK/54/98 H1N1 for explant 5. Data are representative of two independent experiments and normalized to mock infected controls.

In the next series of experiments, using the non-tumor human lung explant tissues, we examined the effects of an IFN-α, namely IFN alfacon-1, on H5N1 and H1N1 influenza A infection. Human lung explant tissues were pretreated with IFN-alfacon-1 for 16 hours prior to infection with H5N1 or H1N1 influenza A viruses. At different time points post infection, RNA was extracted for cDNA synthesis. Analysis of influenza A *m* gene expression revealed that IFN alfacon-1 treatment effectively inhibits H5N1 and H1N1 influenza A replication (Figure 6A). Gene expression analysis for 2′5′-*oas*, *pkr* and *isg15*, IFN-stimulated genes (ISGs) associated with an IFN-inducible antiviral response, revealed that the expression levels for these ISGs were not upregulated in H5N1 or H1N1 virus infected tissues. However, their expression was upregulated in both H5N1 and H1N1 virus infected human lung tissue treated with IFN alfacon-1 (Figure 6B).

**Figure 7:**
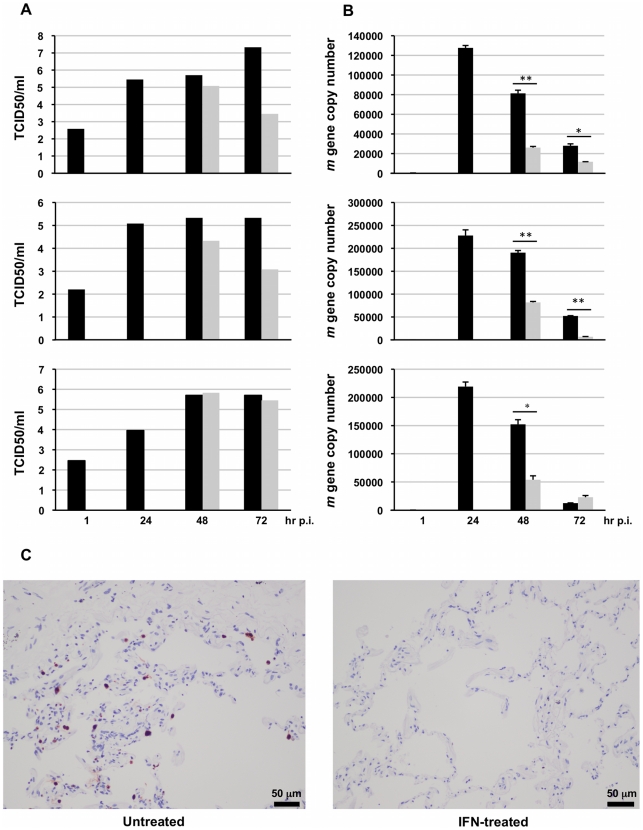
Treatment with IFN alfacon-1 inhibits *pandemic* H1N1 2009 virus replication in human lung tissue. Three different human surgical lung tissue explants were infected with pandemic H1N1 2009 virus for 24 hr, then either left untreated (▪) or treated with IFN alfacon-1 (1.2×104 U/mL) (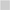) for a further 48 hr. At the indicated times **A**) Viral titers (TCID_50_) and **B**) Influenza A *m* gene expression were measured; Significant differences were determined by Student t-test: * ∼ p<0.05; ** ∼ p<0.01. **C**) Thin sections of infected human lung explants, either untreated (i) or IFN-treated (ii) were stained for influenza A nucleoprotein (pink).

In a final series of experiments we examined the effects of IFN alfacon-1 on pandemic H1N1 influenza A infection, when IFN was added post-challenge with virus. Three different human lung explants were infected with H1N1pdm virus, then 24 hours post-infection treated with 1.2×10^4^ U/ml IFN alfacon-1. At 24 and 48 hours post-treatment, the effects of IFN on viral replication were evaluated by measuring *m* gene expression and TCID_50_ values. The results in Figure 7, panels A and B, provide evidence for the protective effects of IFN treatment, even when added postinfection, as assessed by TCID_50_ and *m* gene expression. These results are supported by evidence of a reduction in Influenza A nucleoprotein expression, visualized in the IFN-treated lung explants (Figure 7C).

## Discussion

Prompted by an increase of oseltamivir-resistant influenza A virus isolates, this study was initiated to determine the effect of NS1 on IFN signaling, and to explore the therapeutic potential of IFN to override any NS1-mediated inhibitory effects. Any IFN response is augmented by a positive feedback loop; specifically, IFN produced in response to virus infection binds to and activates IFNAR, leading to gene induction, which includes IFN. Thus IFN exerts both direct and paracrine effects on producer and adjacent cells. Previous studies indicate that H5N1 is able to overcome this feedback loop and the NS1 of H5N1 has been implicated in the development of this resistance [22].

Herein we report novel strategies by which the H5N1 influenza virus NS1 alters IFN-α/β signaling, mediated by the inhibition of IFNAR and SOCS protein expression. The inhibitory effects of NS1 on IFNs-α/β have largely been attributed to its ability to inhibit IFN induction. A number of viruses have evolved to target STAT proteins and block the antiviral activity of IFNs: Paramyxoviruses such as SV5 and type II human parainfluenza viruses (HPIV2) block IFN signaling through their V proteins, which induce proteasomal degradation of STAT1 and STAT2 by polyubiquitation [23]. HCV core proteins block STAT1 activation and subsequent function, mediated by STAT1-core protein interactions and suppression of STAT1 gene expression [24]. We provide evidence that expression of the H5N1 influenza virus NS1 in HeLa cells leads to a reduction of IFN-inducible STAT phosphorylation (Figure 1).

The phosphorylation-dependent activation of STAT1 and STAT2 is critical for mediating IFN-inducible antiviral responses [25]. Activated STAT proteins form various complexes that subsequently translocate into the nucleus to initiate gene expression via binding to specific elements in the promoter regions of ISGs. In the absence of these transcriptional effector proteins, cells are unresponsive to IFNs and are highly susceptible to viral infection [26], [27]. The inhibition of IFN-inducible phosphorylation of STATs in the presence of H5N1 NS1 resulted in a reduction in the formation of the characteristic STAT1:1:SIE, STAT1:3:SIE and STAT3:3:SIE complexes (Figure 2).

These results prompted us to evaluate whether NS1 might affect upstream effectors of the STATs, thereby limiting their IFN-inducible phosphorylation and activation. FACS and confocal microscopy analysis of surface IFNAR1 and IFNAR2 expression revealed a reduction in cell surface IFNAR1 in the presence of H5N1 NS1, yet IFNAR2 surface expression remained unaltered (Figure 3). Based on the ability of NS1 to inhibit host mRNA generation, we examined NS1 effects on *ifnar1* and *ifnar2* gene expression, and showed that after an initial, early increase in *ifnar1* and *ifnar2* expression, likely reflective of an innate cellular response, there was a reduction in both *ifnar1* and *ifnar2* expression in monocyte-derived macrophages by 24 hours, but a more selective reduction in expression of *ifnar1* in *ex vivo* lung tissues by 16 hours, once viral replication became fully established (Figure 4). This NS1-dependent reduction in *ifnar1* gene expression is likely responsible for the decrease in IFN-inducible STAT phosphorylation and DNA binding. The basal level of expression of distinct signaling effectors, including IFNAR1, JAK1, TYK2, IRF9 and STAT2, has been shown to correlate directly with the intensity of an IFN response [28]. IFNAR1 null cells are completely non-responsive to IFN treatment and mice null for IFNAR1 are, likewise, unresponsive to IFNs-α/β and highly susceptible to microbial infections [29]. Clinical studies of HCV patients whom are either non-responders or exhibit a reduced sensitivity to IFN therapy, identified a reduction in either *ifnar1* or *ifnar2* gene expression when compared to IFN responders [30]. Polymorphisms in the promoter region of *ifnar1* and *ifnar2* have been closely linked with susceptibility to a number of diseases including malaria, multiple sclerosis, trypanosomaiasis, HCV and HIV [31], [32], [33], [34]. In contrast, over-expression of IFNAR1 and IFNAR2, as is the case in Down's Syndrome patients, where chromosome 21 is trisomic, results in an enhanced sensitivity to IFN [35].

While the specific mechanism by which H5N1 NS1 downregulates IFNAR1 expression requires further investigation, NS1 will inhibit pre-mRNA splicing, polyadenylation and nuclear export. NS1 interacts with components of the splicing machinery, U6 snRNA, which complex with other constituents of the spliceosome to mediate pre-mRNA splicing [11]. An association between NS1 and U6 snRNA hinders its ability to complex with other catalytic subunits of the spliceosome, thereby leading to the accumulation of pre-mRNAs in the nucleus of the host cell. Additionally, NS1 affects polyadenylation of host mRNA through targeting of CPSF30 and PABII [36], [37]. 3′ cleavage and polyadenylation of mRNAs promotes their export into the cytoplasm, whereas mRNAs that have undergone 3′ cleavage alone are retained in the nucleus [38]. Viewed together, the inhibitory effect of influenza virus H5N1 NS1 on *ifnar1* gene expression is an effective mechanism to render target cells insensitive to IFN and overrides the initial non-specific antiviral effect of IFNAR upregulation seen in the monocyte derived macrophages.

IFNs-α/β are not only critical components of the innate immune response, but also play a prominent role in modulating an adaptive immune response. IFNs-α promote the differentiation and maturation of dendritic cells (DCs), which subsequently present viral peptide in the context of the major histocompatibility complex (MHC) to activate T cells. Additionally, IFNs-α/β can modulate co-stimulatory molecule expression, to further stimulate or prime virus-specific CD4^+^ and CD8^+^ T cells. During influenza A virus infection, expression of NS1 in DCs blocks their maturation and subsequently results in ineffective T cell activation [39]. NS1 expression in DCs alters the expression of numerous genes that are required for both maturation and migration, including *ifnar1* [39].

Notably, infection with the H5N1 influenza A strain led to a greater reduction in *ifnar1* gene expression compared to infection with H1N1 virus (Figure 4C, D) in the *ex vivo* lung tissue model. X-ray crystallographic studies suggest that NS1 from highly pathogenic H5N1 influenza A virus exhibits structural differences in both the RNA-binding and effector domains when compared to NS1 from other influenza strains. H5N1 NS1 can associate with itself to form a novel tubular oligomeric structure, whereas NS1 from other strains adopt a dimeric conformation [40], [41]. These conformational differences may contribute to the different degrees of inhibition of *ifnar1* gene expression observed between H5N1 and H1N1.

SOCS proteins are potent inhibitors of JAK/STAT signaling. There is accumulating evidence that influenza virus infection will also inhibit an IFN response in part through up-regulation of SOCS1 and/or SOCS3 expression [42], [43]. SOCS1 inhibits IFN signaling through direct physical interactions with JAK1, whereas SOC3 and CIS interact with the phosphorylated receptor to hinder the recruitment and phosphorylation of downstream effectors such as STATs [44], [45]. Over-expression of SOCS1 and/or SOCS3 will effectively reduce IFN responses through the inhibition of STAT phosphorylation and induction of ISGs [46]. Many viruses, including respiratory syncytial virus, herpesviruses and hepatitis C virus, modulate SOCS1 expression to inhibit STAT activation as a way to suppress an IFN response [47], [48], [49]. Our data revealed that both *socs1* and *socs3* gene expression increased in HeLa cells expressing H5N1 NS1, yet we were only able to detect an increase in SOCS1 protein expression (Figure 5). SOCS1 expression can be induced by various cytokines in a tissue-specific manner [50], [51]. STAT proteins including STAT1 and STAT5 have been suggested to play a role in mediating the transcriptional activation of *socs* gene expression upon cytokine stimulation [50], [51]. Studies in breast cancer cells suggest that MAP kinase (MAPK) p38 activation, an intermediate effector in MAPK signaling, may also play a role in upregulating SOCS1 expression. The hyperinduction of proinflammatory cytokines in H5N1 but not H1N1 influenza A-infected primary human macrophages was strongly associated with p38 activation, suggesting a possible mechanism for the induction of SOCS1 expression [52], [53], [54]. Notably, SOCS1 expression is regulated at the translational level in a cap-dependent manner by the eukaryotic initiation factor 4E-binding proteins [55]. NS1 affects cellular translation by interacting with eIF4G and activating the PI3K pathway [12], [13], [56], [57]. Activation of PI3K can lead to translational activation through mTOR and subsequent phosphorylation of 4E-BP1, ultimately leading to an increase in cap-dependent mRNA translation [58].

The adamantine derivatives, amantadine and rimantadine, as M2 ion channel-blockers were the first antivirals licensed for use against influenza A viruses [59], yet all isolates of the pandemic H1N1 2009 are resistant to both amantadine and rimantadine [60]. There are two other drugs licensed globally for specific treatment and prevention of influenza. Relenza (zanamavir) was developed based on knowledge of the 3-dimensional structure of the influenza virus NA complexed with its substrate, sialic acid [61]. Tamiflu (oseltamivir) was subsequently developed, based on the structure of Relenza [62]. Relenza has poor bioavailability, it must be delivered topically and is administered by means of an inhaler, which delivers drug to the upper respiratory tract, the primary site of virus replication. Substitution of the glycerol side chain with a hydrophobic side chain enables Tamiflu to be orally available. Tamiflu is taken in an encapsulated form as a prodrug, which is activated by liver esterases to form the active drug [63]. Although the pandemic H1N1 2009 virus is sensitive to oseltamivir, resistance has been detected in isolated clinical cases in Hong Kong, Denmark, Japan and Canada [64]. For H5N1 virus, reports of drug resistance in approximately 2% of adult patients and 18% of pediatric patients infected, raises concerns [65].

In both guinea pig and ferret models, IFN-α treatment effectively inhibits both H1N1 and H5N1 viral replication [22], [66], though multiple doses appear necessary in the guinea pig model. Viewed together with the evidence we provide for the antiviral effects of IFN against H1N1 and H5N1 in the human lung explant tissue model, we infer that despite the inhibitory mechanisms employed by NS1 to target an IFN response, IFN treatment can override these effects (Figure 6). Notably, IFN treatment was inhibitory against both H5N1 and H1N1 influenza A strains, including the pandemic H1N1 influenza 2009. Moreover, we provide evidence that IFN treatment *post-challenge* with virus is effective at limiting viral replication: human lung explants when infected with H1N1pdm exhibited IFN-inducible reduction in viral titer, M gene and influenza nucleoprotein expression. Given the broad spectrum antiviral activities of IFNs, that are not pathogen-specific, development of resistance is avoided. Accordingly, in ongoing studies we are evaluating the safety profile and therapeutic potential of IFN alfacon-1 treatment in individuals infected with influenza-like illness, specifically pandemic H1N1pdm.

## References

[1] Saito T, Gale M (2007). Principles of intracellular viral recognition.. Curr Opin Immunol.

[2] Bonjardim CA, Ferreira PC, Kroon EG (2009). Interferons: signaling, antiviral and viral evasion.. Immunol Lett.

[3] Hengel H, Koszinowski UH, Conzelmann KK (2005). Viruses know it all: new insights into IFN networks.. Trends Immunol.

[4] Hale BG, Randall RE, Ortin J, Jackson D (2008). The multifunctional NS1 protein of influenza A viruses.. J Gen Virol.

[5] Guo Z, Chen LM, Zeng H, Gomez JA, Plowden J (2007). NS1 protein of influenza A virus inhibits the function of intracytoplasmic pathogen sensor, RIG-I.. Am J Respir Cell Mol Biol.

[6] Pichlmair A, Schulz O, Tan CP, Naslund TI, Liljestrom P (2006). RIG-I-mediated antiviral responses to single-stranded RNA bearing 5′-phosphates.. Science.

[7] Mibayashi M, Martinez-Sobrido L, Loo YM, Cardenas WB, Gale M (2007). Inhibition of retinoic acid-inducible gene I-mediated induction of beta interferon by the NS1 protein of influenza A virus.. J Virol.

[8] Gack MU, Albrecht RA, Urano T, Inn KS, Huang IC (2009). Influenza A virus NS1 targets the ubiquitin ligase TRIM25 to evade recognition by the host viral RNA sensor RIG-I.. Cell Host Microbe.

[9] Wang X, Li M, Zheng H, Muster T, Palese P (2000). Influenza A virus NS1 protein prevents activation of NF-kappaB and induction of alpha/beta interferon.. J Virol.

[10] Donelan NR, Dauber B, Wang X, Basler CF, Wolff T (2004). The N- and C-terminal domains of the NS1 protein of influenza B virus can independently inhibit IRF-3 and beta interferon promoter activation.. J Virol.

[11] Qiu Y, Nemeroff M, Krug RM (1995). The influenza virus NS1 protein binds to a specific region in human U6 snRNA and inhibits U6-U2 and U6-U4 snRNA interactions during splicing.. RNA.

[12] Shin YK, Li Y, Liu Q, Anderson DH, Babiuk LA (2007). SH3 binding motif 1 in influenza A virus NS1 protein is essential for PI3K/Akt signaling pathway activation.. J Virol.

[13] Ehrhardt C, Wolff T, Pleschka S, Planz O, Beermann W (2007). Influenza A virus NS1 protein activates the PI3K/Akt pathway to mediate antiapoptotic signaling responses.. J Virol.

[14] Bright RA, Medina MJ, Xu X, Perez-Oronoz G, Wallis TR (2005). Incidence of adamantane resistance among influenza A (H3N2) viruses isolated worldwide from 1994 to 2005: a cause for concern.. Lancet.

[15] Wang SQ, Du QS, Huang RB, Zhang DW, Chou KC (2009). Insights from investigating the interaction of oseltamivir (Tamiflu) with neuraminidase of the 2009 H1N1 swine flu virus.. Biochem Biophys Res Commun.

[16] Cheng PK, Leung TW, Ho EC, Leung PC, Ng AY (2009). Oseltamivir- and amantadine-resistant influenza viruses A (H1N1).. Emerg Infect Dis.

[17] Lee SM, Cheung CY, Nicholls JM, Hui KP, Leung CY (2008). Hyperinduction of cyclooxygenase-2-mediated proinflammatory cascade: a mechanism for the pathogenesis of avian influenza H5N1 infection.. J Infect Dis.

[18] Karber G (1931). 50% end-point calculation.. Arch Exp Pathol Pharmak.

[19] Goldman LA, Zafari M, Cutrone EC, Dang A, Brickelmeier M (1999). Characterization of antihuman IFNAR-1 monoclonal antibodies: epitope localization and functional analysis.. J Interferon Cytokine Res.

[20] Brierley MM, Marchington KL, Jurisica I, Fish EN (2006). Identification of GAS-dependent interferon-sensitive target genes whose transcription is STAT2-dependent but ISGF3-independent.. FEBS J.

[21] Rahbar R, Murooka TT, Hinek AA, Galligan CL, Sassano A (2006). Vaccinia virus activation of CCR5 invokes tyrosine phosphorylation signaling events that support virus replication.. J Virol.

[22] Van Hoeven N, Belser JA, Szretter KJ, Zeng H, Staeheli P (2009). Pathogenesis of 1918 pandemic and H5N1 influenza virus infections in a guinea pig model: antiviral potential of exogenous alpha interferon to reduce virus shedding.. J Virol.

[23] Precious B, Young DF, Andrejeva L, Goodbourn S, Randall RE (2005). In vitro and in vivo specificity of ubiquitination and degradation of STAT1 and STAT2 by the V proteins of the paramyxoviruses simian virus 5 and human parainfluenza virus type 2.. J Gen Virol.

[24] Lin W, Kim SS, Yeung E, Kamegaya Y, Blackard JT (2006). Hepatitis C virus core protein blocks interferon signaling by interaction with the STAT1 SH2 domain.. J Virol.

[25] Dupuis S, Jouanguy E, Al-Hajjar S, Fieschi C, Al-Mohsen IZ (2003). Impaired response to interferon-alpha/beta and lethal viral disease in human STAT1 deficiency.. Nat Genet.

[26] Park C, Li S, Cha E, Schindler C (2000). Immune response in Stat2 knockout mice.. Immunity.

[27] Durbin JE, Hackenmiller R, Simon MC, Levy DE (1996). Targeted disruption of the mouse Stat1 gene results in compromised innate immunity to viral disease.. Cell.

[28] Zurney J, Howard KE, Sherry B (2007). Basal expression levels of IFNAR and Jak-STAT components are determinants of cell-type-specific differences in cardiac antiviral responses.. J Virol.

[29] Hwang SY, Hertzog PJ, Holland KA, Sumarsono SH, Tymms MJ (1995). A null mutation in the gene encoding a type I interferon receptor component eliminates antiproliferative and antiviral responses to interferons alpha and beta and alters macrophage responses.. Proc Natl Acad Sci U S A.

[30] Welzel TM, Morgan TR, Bonkovsky HL, Naishadham D, Pfeiffer RM (2009). Variants in interferon-alpha pathway genes and response to pegylated interferon-Alpha2a plus ribavirin for treatment of chronic hepatitis C virus infection in the hepatitis C antiviral long-term treatment against cirrhosis trial.. Hepatology.

[31] Tena-Tomas C, Pedroso ML, de Messias-Reason IJ, Kremsner PG, Kun JF (2007). Polymorphisms in the IFNAR1 gene in patients with chronic hepatitis C: outcome of combined IFN-alpha therapy.. Eur Cytokine Netw.

[32] Diop G, Hirtzig T, Do H, Coulonges C, Vasilescu A (2006). Exhaustive genotyping of the interferon alpha receptor 1 (IFNAR1) gene and association of an IFNAR1 protein variant with AIDS progression or susceptibility to HIV-1 infection in a French AIDS cohort.. Biomed Pharmacother.

[33] Aucan C, Walley AJ, Hennig BJ, Fitness J, Frodsham A (2003). Interferon-alpha receptor-1 (IFNAR1) variants are associated with protection against cerebral malaria in the Gambia.. Genes Immun.

[34] Leyva L, Fernandez O, Fedetz M, Blanco E, Fernandez VE (2005). IFNAR1 and IFNAR2 polymorphisms confer susceptibility to multiple sclerosis but not to interferon-beta treatment response.. J Neuroimmunol.

[35] Mowshowitz SL, Dawson GJ, Elizan TS (1983). Antiviral response of fibroblasts from familial Alzheimer's disease and Down's syndrome to human interferon-alpha.. J Neural Transm.

[36] Nemeroff ME, Barabino SM, Li Y, Keller W, Krug RM (1998). Influenza virus NS1 protein interacts with the cellular 30 kDa subunit of CPSF and inhibits 3′end formation of cellular pre-mRNAs.. Mol Cell.

[37] Chen Z, Li Y, Krug RM (1999). Influenza A virus NS1 protein targets poly(A)-binding protein II of the cellular 3′-end processing machinery.. EMBO J.

[38] Fuke H, Ohno M (2008). Role of poly (A) tail as an identity element for mRNA nuclear export.. Nucleic Acids Res.

[39] Fernandez-Sesma A, Marukian S, Ebersole BJ, Kaminski D, Park MS (2006). Influenza virus evades innate and adaptive immunity via the NS1 protein.. J Virol.

[40] Wang W, Riedel K, Lynch P, Chien CY, Montelione GT (1999). RNA binding by the novel helical domain of the influenza virus NS1 protein requires its dimer structure and a small number of specific basic amino acids.. RNA.

[41] Bornholdt ZA, Prasad BV (2008). X-ray structure of NS1 from a highly pathogenic H5N1 influenza virus.. Nature.

[42] Pauli EK, Schmolke M, Wolff T, Viemann D, Roth J (2008). Influenza A virus inhibits type I IFN signaling via NF-kappaB-dependent induction of SOCS-3 expression.. PLoS Pathog.

[43] Pothlichet J, Chignard M, Si-Tahar M (2008). Cutting edge: innate immune response triggered by influenza A virus is negatively regulated by SOCS1 and SOCS3 through a RIG-I/IFNAR1-dependent pathway.. J Immunol.

[44] Matsumoto A, Seki Y, Kubo M, Ohtsuka S, Suzuki A (1999). Suppression of STAT5 functions in liver, mammary glands, and T cells in cytokine-inducible SH2-containing protein 1 transgenic mice.. Mol Cell Biol.

[45] Yasukawa H, Misawa H, Sakamoto H, Masuhara M, Sasaki A (1999). The JAK-binding protein JAB inhibits Janus tyrosine kinase activity through binding in the activation loop.. EMBO J.

[46] Vlotides G, Sorensen AS, Kopp F, Zitzmann K, Cengic N (2004). SOCS-1 and SOCS-3 inhibit IFN-alpha-induced expression of the antiviral proteins 2,5-OAS and MxA.. Biochem Biophys Res Commun.

[47] Oshansky CM, Krunkosky TM, Barber J, Jones LP, Tripp RA (2009). Respiratory syncytial virus proteins modulate suppressors of cytokine signaling 1 and 3 and the type I interferon response to infection by a toll-like receptor pathway.. Viral Immunol.

[48] Frey KG, Ahmed CM, Dabelic R, Jager LD, Noon-Song EN (2009). HSV-1-Induced SOCS-1 Expression in Keratinocytes: Use of a SOCS-1 Antagonist to Block a Novel Mechanism of Viral Immune Evasion..

[49] Bode JG, Ludwig S, Ehrhardt C, Albrecht U, Erhardt A (2003). IFN-alpha antagonistic activity of HCV core protein involves induction of suppressor of cytokine signaling-3.. FASEB J.

[50] Krebs DL, Hilton DJ (2000). SOCS: physiological suppressors of cytokine signaling.. J Cell Sci.

[51] Chen XP, Losman JA, Rothman P (2000). SOCS proteins, regulators of intracellular signaling.. Immunity.

[52] Park Y, Shon SK, Kim A, Kim KI, Yang Y (2007). SOCS1 induced by NDRG2 expression negatively regulates STAT3 activation in breast cancer cells.. Biochem Biophys Res Commun.

[53] Hui KP, Lee SM, Cheung CY, Ng IH, Poon LL (2009). Induction of proinflammatory cytokines in primary human macrophages by influenza A virus (H5N1) is selectively regulated by IFN regulatory factor 3 and p38 MAPK.. J Immunol.

[54] Chan MC, Cheung CY, Chui WH, Tsao SW, Nicholls JM (2005). Proinflammatory cytokine responses induced by influenza A (H5N1) viruses in primary human alveolar and bronchial epithelial cells.. Respir Res.

[55] Gregorieff A, Pyronnet S, Sonenberg N, Veillette A (2000). Regulation of SOCS-1 expression by translational repression.. J Biol Chem.

[56] Burgui I, Aragon T, Ortin J, Nieto A (2003). PABP1 and eIF4GI associate with influenza virus NS1 protein in viral mRNA translation initiation complexes.. J Gen Virol.

[57] Aragon T, de la Luna S, Novoa I, Carrasco L, Ortin J (2000). Eukaryotic translation initiation factor 4GI is a cellular target for NS1 protein, a translational activator of influenza virus.. Mol Cell Biol.

[58] Asnaghi L, Bruno P, Priulla M, Nicolin A (2004). mTOR: a protein kinase switching between life and death.. Pharmacol Res.

[59] Jefferson T, Deeks JJ, Demicheli V, Rivetti D, Rudin M (2004). Amantadine and rimantadine for preventing and treating influenza A in adults.. Cochrane Database Syst Rev.

[60] Cheung CL, Rayner JM, Smith GJ, Wang P, Naipospos TS (2006). Distribution of amantadine-resistant H5N1 avian influenza variants in Asia.. J Infect Dis.

[61] von Itzstein M, Thomson R (2009). Anti-influenza drugs: the development of sialidase inhibitors.. Handb Exp Pharmacol.

[62] Kim CU, Lew W, Williams MA, Liu H, Zhang L (1997). Influenza neuraminidase inhibitors possessing a novel hydrophobic interaction in the enzyme active site: design, synthesis, and structural analysis of carbocyclic sialic acid analogues with potent anti-influenza activity.. J Am Chem Soc.

[63] Moscona A (2005). Neuraminidase inhibitors for influenza.. N Engl J Med.

[64] CDC (2009). Oseltamivir-Resistant Novel Influenza A (H1N1) Virus Infection in Two Immunosuppressed Patients.. MMWR.

[65] Hill AW, Guralnick RP, Wilson MJ, Habib F, Janies D (2009). Evolution of drug resistance in multiple distinct lineages of H5N1 avian influenza.. Infect Genet Evol.

[66] Kugel D, Kochs G, Obojes K, Roth J, Kobinger GP (2009). Intranasal administration of alpha interferon reduces seasonal influenza A virus morbidity in ferrets.. J Virol.

